# Infrared inhibition impacts on locally initiated and propagating action potentials and the downstream synaptic transmission

**DOI:** 10.1117/1.NPh.7.4.045003

**Published:** 2020-10-14

**Authors:** Xuedong Zhu, Jen-Wei Lin, Michelle Y. Sander

**Affiliations:** a Boston University, Department of Biomedical Engineering, Boston, Massachusetts, United States; b Boston University, Neurophotonics Center, Boston, Massachusetts, United States; c Boston University, Photonics Center, Boston, Massachusetts, United States; d Boston University, Department of Biology, Boston, Massachusetts, United States; e Boston University, Department of Electrical and Computer Engineering, Boston, Massachusetts, United States; f Boston University, Division of Materials Science and Engineering, Brookline, Massachusetts, United States

**Keywords:** infrared nerve inhibition, neural modulation, synaptic transmission, action potential initiation, action potential propagation, photothermal effect

## Abstract

**Significance:** Systematic studies of the physiological outputs induced by infrared (IR)-mediated inhibition of motor nerves can provide guidance for therapeutic applications and offer critical insights into IR light modulation of complex neural networks.

**Aim:** We explore the IR-mediated inhibition of action potentials (APs) that either propagate along single axons or are initiated locally and their downstream synaptic transmission responses.

**Approach:** APs were evoked locally by two-electrode current clamp or at a distance for propagating APs. The neuromuscular transmission was recorded with intracellular electrodes in muscle cells or macro-patch pipettes on terminal bouton clusters.

**Results:** IR light pulses completely and reversibly terminate the locally initiated APs firing at low frequencies, which leads to blocking of the synaptic transmission. However, IR light pulses only suppress but do not block the amplitude and duration of propagating APs nor locally initiated APs firing at high frequencies. Such suppressed APs do not influence the postsynaptic responses at a distance. While the suppression of AP amplitude and duration is similar for propagating and locally evoked APs, only the former exhibits a 7% to 21% increase in the maximum time derivative of the AP rising phase.

**Conclusions:** The suppressed APs of motor axons can resume their waveforms after passing the localized IR light illumination site, leaving the muscular and synaptic responses unchanged. IR-mediated modulation on propagating and locally evoked APs should be considered as two separate models for axonal and somatic modulations.

## 1 Introduction

Infrared (IR) light has emerged as a new modality that can reliably modulate both neural and muscular activities with the advantages of being contact-free, spatially selective, and MRI compatible.^1^^–^^3^ IR-mediated modulation of excitable tissues has been demonstrated in a wide range of promising clinical applications, such as cochlear prostheses,^4^^,^^5^ brain stimulation and mapping,^6^^–^^9^ cardiac pacing,^10^ and neural monitoring during surgery.^11^^,^^12^ The main biological processes induced by pulsed IR light are attributed to the spatiotemporal thermal transients generated by water and tissue absorption,^13^ which in turn can alter the membrane capacitance,^14^^–^^17^ membrane resistance,^18^^,^^19^ ion channel activities,^19^^–^^25^ and intracellular calcium dynamics.^6^^,^^26^^–^^30^ The temperature-sensitive transient receptor potential channels have received particular attention due to their intrinsic temperature sentisitivity.^25^^,^^27^^,^^28^^,^^30^^,^^31^ The presence of multiple IR-interacting targets in neural tissues suggests the need for an integrated approach to understand IR-mediated modulation. One unresolved question is which of the mentioned targets dictate a net dominant functional output in a preparation for given experimental conditions. Furthermore, the overall system response needs to be understood in how these targets not only individually but collectively modulate neural functions. At the single-neuron level, the function of a neuron as a whole that has been altered by IR light, including its intrinsic excitability, integration of synaptic inputs, and downstream synaptic outputs, has yet to be examined in depth. At the upper-most level, the response of a brain region to IR light modulation will rely on the net result from the interaction of IR light with excitatory and inhibitory components of the network.^9^ Gaining insights into how a neuron as a functional whole is modulated by IR light irradiation is the first step toward a more thorough understanding of IR-mediated modulation of a complex network.

The inhibition of excitable tissues,^19^^–^^21^^,^^32^^–^^36^ including both neural and cardiac preparations, by IR light pulses has been studied extensively and is commonly referred to as IR nerve inhibition (INI). The consensus is that the INI results from temperature-dependent changes of passive membrane properties as well as sodium and potassium ion channel kinetics.^19^^–^^24^ Due to the precise localization of IR light application and the size of experimental animals, most studies using IR light to inhibit peripheral nerves target a relatively small portion of the nerve.^19^^–^^21^^,^^24^ Moreover, IR light application to peripheral nerves involves intercepting propagating APs that are depolarized by a strong charging current and typically take off from a resting membrane potential. However, the consideration of peripheral nerve inhibition is significantly different from the application of IR light to a mammalian central nervous system. The latter involves IR light interactions with the neuronal soma and the initial axonal segments, where the AP initiation occurs when the summation of synaptic potentials crosses the firing threshold. This happens at a time scale significantly slower than propagating APs.^37^^,^^38^ A modeling study has suggested that higher IR light power is required to block conducting APs than APs evoked locally.^22^ The nature and efficacy of IR-mediated neural modulation of these two types of APs have not been compared experimentally. In addition, while there have been many studies demonstrating IR light-mediated inhibition of APs in peripheral nerves, relatively few have investigated the resulting motor output.^20^ Modeling research^22^^,^^23^ showed that partially suppressed APs can resume the full waveform after leaving the localized area with elevated temperatures, while it may require a transient temperature rise of 20°C to 30°C to completely block propagating APs in single axons.^22^^,^^23^ Thus, the goal of achieving motor modulation by IR light irradiation of motor nerves requires a balance between the inhibition of physiological outcomes and minimizing thermal damage to the target tissue. For potential applications of IR light modulation *in vivo*, these questions should be examined systematically and investigated in the context of INI of individual neural tissues.

In this paper, we use the crayfish opener neuromuscular preparation as a model to address the two issues mentioned already by examining the IR-mediated inhibition on locally initiated and propagating APs in motor axons. Synaptic transmission controlled by APs inhibited by IR light is examined by postsynaptic recordings from muscle cells and terminal bouton clusters. We use the APs generated by two intracellular electrodes placed close together in a two-electrode current clamp (TECC) configuration to approximate the IR light inhibition effects on AP firing initiated by a synaptic drive (${\mathrm{AP}}_{\mathrm{TECC}}$). In the second configuration, we examine the IR light inhibition effects on propagating APs (${\mathrm{AP}}_{\mathrm{prop}}$) by placing an extracellular stimulating electrode far away from the intracellular recording electrode. We study the functional relevance of the IR-mediated inhibition on motor axons by examining their synaptic outputs. A precise account for these processes with both intracellular and extracellular recordings reported here can provide valuable insights into IR-mediated neural modulation processes in the peripheral and central nervous systems.

## 2 Materials and Methods

### 2.1 Neuromuscular Preparation and Electrophysiological Recording

Crayfish (*Procambarus clarkii*) of both sexes were purchased from Niles Biological Supplies (Sacramento, California). The opener neuromuscular preparations from the first pair of walking legs were dissected in a 35-mm petri dish containing physiological saline (mM): 195 NaCl, 5.4 KCl, 13.5 ${\mathrm{CaCl}}_{2}$, 2.6 ${\mathrm{MgCl}}_{2}$, and 10 HEPES (pH 7.4). The saline was circulated by a peristaltic pump (Cole-Parmer, Illinois) at a rate of 1 to 1.5  ml/min during recording. The axons were separated from the central nerve system so there was no spontaneous axonal activity. Both the inhibitory and the excitatory motor axons were used in this study. Inhibitory axons were used in TECC studies to minimize possible muscle contraction when axons were fired at high frequencies. Excitatory axons were used in ${\mathrm{AP}}_{\mathrm{prop}}$ and macro-patch recordings to take the advantage of higher signal-to-noise ratios of excitatory postsynaptic potentials (EPSPs) and excitatory postsynaptic currents (EPSCs). To obtain a consistent and stable AP firing, 4-aminopyridine (4-AP) at 100 to 200  μM was used in some preparations to block part of the low threshold $K^{+}$ channels.^39^ Recordings were started 20 min after 4-AP was added to ensure steady levels of blockage. Intracellular recordings from the axon used microelectrodes filled with 500-mM KCl (40 to 60  MΩ), while microelectrodes filled with 3 M KCl (10 to 20  MΩ) were used for intracellular recordings from muscle cells.

**Fig. 1:**
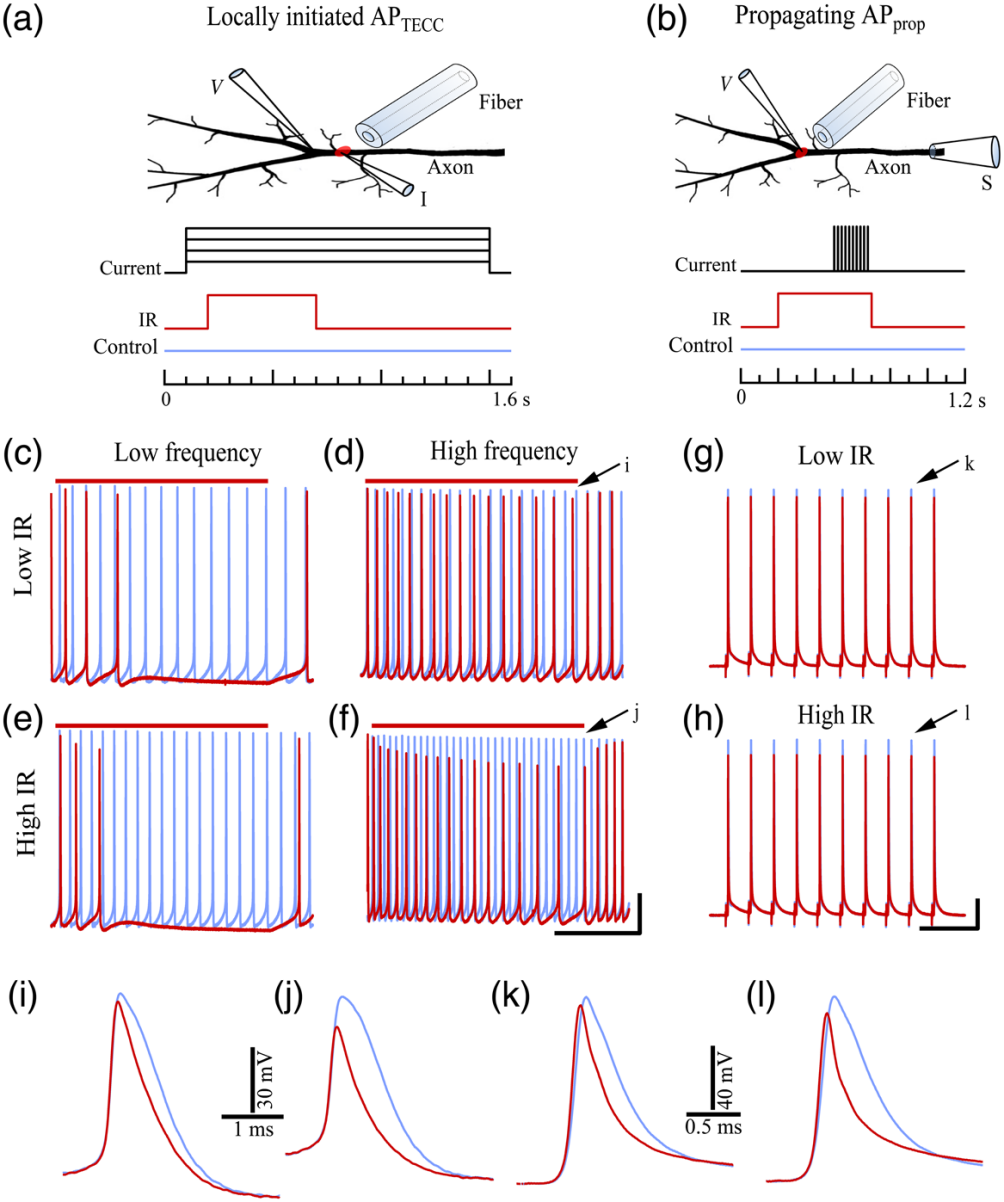
IR-mediated block of locally initiated APs (${\mathrm{AP}}_{\mathrm{TECC}}$) and suppression of propagating APs (${\mathrm{AP}}_{\mathrm{prop}}$). (a) and (b) Schematics of the experimental configuration and protocol to evaluate the IR-mediated inhibition of (a) ${\mathrm{AP}}_{\mathrm{TECC}}$ and (b) ${\mathrm{AP}}_{\mathrm{prop}}$. (c) and (d) ${\mathrm{AP}}_{\mathrm{TECC}}$ trains at low and high firing frequencies evoked by 14- and 18-nA current steps, with (red) and without (blue) 7.1-mW IR light illumination. (e) and (f) ${\mathrm{AP}}_{\mathrm{TECC}}$ trains at different firing frequencies evoked by 13- and 18-nA current steps, with (red) and without (blue) 13.1-mW IR illumination. Red bars above (c)–(f) indicate the timing of the IR light pulses. The membrane potential of the after hyperpolarization following the first AP in (c)–(f) was about −52  mV. (g) and (h) ${\mathrm{AP}}_{\mathrm{prop}}$ trains activated by extracellular stimulation under (g) 7.1 mW and (h) 13.1-mW IR light illumination. The resting membrane potential was −75  mV. (i)–(l) Zoomed-in versions of the AP pairs indicated by the corresponding arrows in (d), (f), (g), and (h). Calibration: (c)–(f) share the same scale bars as (f) with 20-mV vertical and 200-ms horizontal. (g) and (h) Share the same scale bars as (h) with 20-mV vertical and 50-ms horizontal.

Figures 1(a) and 1(b) show the two experimental configurations and protocols. In Fig. 1(a), TECC was performed with the voltage (V) and current (I) electrodes placed 250 to 300  μm apart, near the main branching point of the inhibitory axon. A series of current steps, from −10 to 30 nA, with 1.4 s in duration, were injected to evoke APs (${\mathrm{AP}}_{\mathrm{TECC}}$) firing, up to 60 Hz. The IR light pulses (500 ms) were applied 100 ms after the onset of the current step.

In the case of propagating APs (${\mathrm{AP}}_{\mathrm{prop}}$), a suction electrode [Fig. 1(b), S] was placed >5  mm upstream to the AP recording site to activate ${\mathrm{AP}}_{\mathrm{prop}}$ in both the excitatory and inhibitory motor axons. The suction electrode stimulation was applied for 200 ms (at 50 Hz), overlapping with the last 200 ms of the IR light pulses, when the IR-induced temperature changes reached a steady state.^19^ The frequency and duration of the suction electrode stimulation were chosen to be comparable to the ${\mathrm{AP}}_{\mathrm{TECC}}$ at high firing frequency and to achieve sufficient synaptic facilitation for detection while avoiding muscle contraction. Postsynaptic recordings from muscle cells were obtained >700  μm distal to the IR light illumination site (see insets in Fig. 4). This separation was to ensure that the recorded muscle cell and synapses were well outside of the localized illumination and temperature rise (Section S1 and Fig. S1 in the Supplementary Material).

The use of the macro-patch technique required visualization of presynaptic terminals. This was achieved by injecting Alexa Fluor 568 (Life Technologies Corporation, California) into the inhibitory axon. Since the terminal varicosities of the excitor and inhibitor appear in pairs on the surface of muscle cells, injection of the fluorescence dye into inhibitory axon allowed for precise localization of excitor terminals.^40^ The macro-patch pipette for the terminal recording^41^ was filled with physiological saline (∼3  MΩ) where the pipette opening featured a diameter of ∼20  μm. This technique also offers good resolution to detect changes in ion channels that shape the AP at the presynaptic terminal.^42^^,^^43^ The recording was performed under an Olympus BX51 microscope with a 60× water immersion lens. The electrophysiological recordings were carried out with AXOCLAMP-2A (Axon Instruments Inc., California), MULTICLAMP 700B (Molecular Devices, California), and IE201 (Warner Instrument Corp., Connecticut). The experiments were conducted at room temperature around 21°C. All chemicals were purchased from Sigma-Aldrich unless specified otherwise.

### 2.2 Infrared Laser Light Configuration

A fiber-coupled diode laser (FPL2000S, Thorlabs) with a wavelength centered at 1994 nm with a 3-dB linewidth of 3.6 nm was used as the illumination source in this study. The delivery fiber with a core diameter of 50  μm was cleaved before each experiment and positioned slightly above the axon surface at an angle of 28° to the horizontal plane. With this configuration, a segment of ∼100  μm of the axon was directly illuminated by the IR light. A red laser diode was coupled into the optical fiber to facilitate the alignment of the invisible IR laser beam. The duration, typically 500 ms, and output power of the IR light were modulated by an Igor Pro (WaveMetrics) software interface and a data acquisition platform (NI USB-6363). The IR light power measured at the delivery end of the fiber pigtail ranged between 7.1 and 13.1 mW. Based on the water absorption and an estimated distance between the axon surface and the fiber tip (∼120  μm), the resulting fluence on the surface of the axon was estimated to be about 11.38 to $21.13\;{J/\mathrm{cm}}^{2}$. The IR-induced temperature transients were monitored with an open patch pipette filled with physiological saline and positioned right above the axon around the center of the IR light illumination.^19^ Single IR light pulses of 500-ms continuous illumination were used for inhibition in this study to facilitate comparison with data published previously.^19^ Furthermore, using a single IR light pulse with 500 ms allowed minimizing the peak power of IR light required since preliminary pulse parameter scanning studies indicated that comparable inhibitory impacts on APs generated by IR light pulse trains at 200 Hz (50% duty cycle) were only obtained when the total energy deposition and temperature rise were similar between these two conditions. The IR-induced temperature rise reached a plateau after ∼200  ms with the stimulation parameters adapted in this study, which also allowed a detailed characterization of APs and synaptic events during the temperature plateau phase of ∼300  ms. The maximum temperature increase was 11.4°C±0.59°C (N=6) for a power of 7.1 mW IR light illumination and 18.6°C±1.5°C (N=4) for 13.1 mW of power (see Fig. S1 in the Supplementary Material).

### 2.3 Data Analysis

Data acquisition and analysis were performed with Igor Pro (WaveMetrics). Voltage signals were filtered at 5 kHz and sampled at 50 kHz (NI USB-6363). Each preparation (N) represented a set of data recorded from the walking leg of a crayfish animal. Statistical results were presented as an average±the standard error of the mean (SEM). Samples with statistically significant differences were tested with the two-tailed Student’s t-test (α=0.05).

## 3 Results

### 3.1 IR Light Pulses Blocked Low-Frequency ${\mathrm{AP}}_{\mathrm{TECC}}$ and Suppressed High-Frequency ${\mathrm{AP}}_{\mathrm{TECC}}$ and ${\mathrm{AP}}_{\mathrm{prop}}$

We first compared the IR-mediated inhibition of the ${\mathrm{AP}}_{\mathrm{TECC}}$ and the ${\mathrm{AP}}_{\mathrm{prop}}$. Figures 1(a) and 1(b) show the two experimental configurations and protocols. IR light pulses with 7.1-mW power delivered close to the current injection electrode blocked the low-frequency ${\mathrm{AP}}_{\mathrm{TECC}}$ initiated by a current step slightly above the firing threshold level [14 nA in Fig. 1(c)]. These IR light pulses similarly reduced the firing frequency but did not silence the firing [Fig. 1(d)] when an 18-nA current step was used to evoke high-frequency firing. IR light illumination with 13.1-mW power generated more pronounced but qualitatively similar inhibition at both low and high firing frequencies, excited by 13- and 18-nA current steps, respectively [Figs. 1(e) and 1(f)]. A detailed comparison of the ${\mathrm{AP}}_{\mathrm{TECC}}$ recorded at the end of the IR light pulse showed that the inhibition of the ${\mathrm{AP}}_{\mathrm{TECC}}$ amplitude and duration was more pronounced for higher power of the IR light illumination [Figs. 1(i) and 1(j)]. For the ${\mathrm{AP}}_{\mathrm{prop}}$, IR light pulses with 7.1- [Fig. 1(g)] and 13.1-mW power [Fig. 1(h)] could not block the ${\mathrm{AP}}_{\mathrm{prop}}$. As with ${\mathrm{AP}}_{\mathrm{TECC}}$, the suppression in ${\mathrm{AP}}_{\mathrm{prop}}$ amplitude and duration also showed the dependence on the power of the IR light illumination [Figs. 1(k) and 1(l)].

**Fig. 2:**
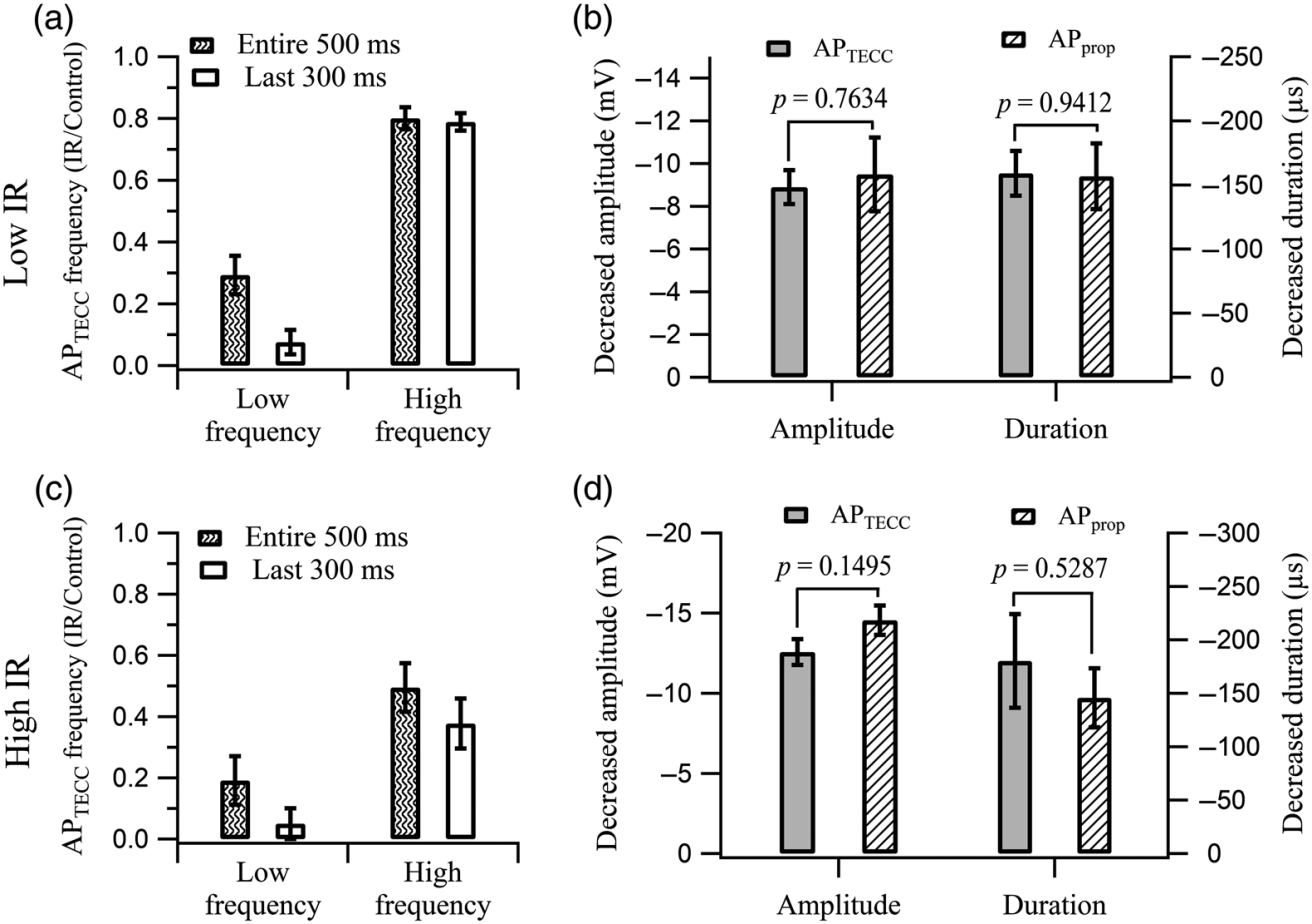
IR light pulses suppressed the ${\mathrm{AP}}_{\mathrm{TECC}}$ firing and inhibited the AP amplitude and duration. (a) IR light pulses with 7.1-mW power significantly reduced the ${\mathrm{AP}}_{\mathrm{TECC}}$ firing at low frequencies (10 to 20 Hz) to 29%±6.2% (N=4) while only decreasing the firing at high frequencies (40 to 60 Hz) to 79%±2.8% (N=8). The ratios of firing frequency calculated from the 500-ms IR light illumination period are presented as the patterned bars. The open bars represent the ratios calculated based on the firing frequency during the last 300 ms of the IR light illumination period, when the temperature rise on the surface of axons had reached steady-state (see Fig. S1 in the Supplementary Material). In this case, the inhibition ratio was 7.5%±3.9% (N=4) for low frequencies and 80%±3.6% (N=8) for high frequencies. (b) IR light with 7.1-mW power suppressed the amplitude and duration of ${\mathrm{AP}}_{\mathrm{TECC}}$ (N=7) and the ${\mathrm{AP}}_{\mathrm{prop}}$ (N=5) to comparable levels. (c) Reduction in ${\mathrm{AP}}_{\mathrm{TECC}}$ firing frequency (N=5) at 13.1-mW IR light pulses illumination. The ${\mathrm{AP}}_{\mathrm{TECC}}$ firing at low frequencies and high frequencies were reduced to 19%±7.9% (N=5) and 49%±8.0% (N=5), respectively, by IR light pulses during the entire 500-ms illumination period. When the firing frequency of ${\mathrm{AP}}_{\mathrm{TECC}}$ during the last 300 ms of the illumination period was evaluated, the inhibition was further reduced to 5.0%±5.0% (N=5) for low frequencies and 38%±8.2% (N=5) for high frequencies. (d) Suppression in amplitude and duration between ${\mathrm{AP}}_{\mathrm{TECC}}$ (N=5) and ${\mathrm{AP}}_{\mathrm{prop}}$ (N=4) was similar when 13.1-mW IR light pulses were used.

To summarize the impact of IR-mediated inhibition on the ${\mathrm{AP}}_{\mathrm{TECC}}$, the frequency of the ${\mathrm{AP}}_{\mathrm{TECC}}$ firing during the entire period of the IR light illumination (500 ms) was calculated and normalized by the average ${\mathrm{AP}}_{\mathrm{TECC}}$ firing frequency during the same period without IR light illumination [Fig. 2(a)]. The ratios were calculated for the low- and high-frequency ranges, with the low-frequency range defined as from 10 to 20 Hz during the control period, while the high-frequency range covered frequencies from 40 to 60 Hz (Fig. S3). The IR light illumination at 7.1 mW reduced the firing frequency of the ${\mathrm{AP}}_{\mathrm{TECC}}$ to 29%±6.2% (N=4) of the control level in the low-frequency range [Fig. 2(a), patterned bar]. Since it took about 200 ms for the IR-induced temperature to rise to steady state^19^ (see Fig. S1 in the Supplementary Material), and the “surviving” 30% of the ${\mathrm{AP}}_{\mathrm{TECC}}$ occurred mainly during this early period [Fig. 1(c)], we also compared the ratio during the last 300 ms of the IR light pulses. In this case, the firing frequency of the ${\mathrm{AP}}_{\mathrm{TECC}}$ was reduced to 7.5%±3.9% (N=4) of the control level [Fig. 2(a), open bar]. For the high firing frequencies evoked by large current steps, the IR light pulses decreased their firing frequency by ∼20% (N=8) regardless of the illumination periods we chose to calculate the ratios [Fig. 2(a)]. When the IR irradiation power was increased from 7.1 to 13.1 mW, the inhibition on ${\mathrm{AP}}_{\mathrm{TECC}}$ firing was more pronounced [Fig. 2(c)]. IR light pulses at 13.1 mW completely blocked the low-frequency ${\mathrm{AP}}_{\mathrm{TECC}}$ firing during the last 300 ms in 4 out of 5 preparations and reduced the firing frequency by ∼60% (N=5) at high firing frequencies.

### 3.2 Similar Inhibitory Effects on the Waveforms of the ${\mathrm{AP}}_{\mathrm{TECC}}$ and ${\mathrm{AP}}_{\mathrm{prop}}$

Although the initiation processes of ${\mathrm{AP}}_{\mathrm{prop}}$ and ${\mathrm{AP}}_{\mathrm{TECC}}$ were different, the inhibitory impacts of IR light pulses on the waveform of these APs were similar. We compared the inhibition in AP amplitude and duration mediated by IR light illumination. To ensure consistency in comparison, only the last pair of APs before the end of the IR light pulses was used for this analysis [Figs. 1(i)–1(l) for example]. The AP amplitude was defined as the difference between the AP peak and the AP firing threshold (dV/dt=25  V/s) and the duration as the full width at half maximum of the AP waveform. The decrease of ${\mathrm{AP}}_{\mathrm{TECC}}$ amplitude (−8.9±0.79  mV, N=7) was statistically indistinguishable (p=0.7634) from that of ${\mathrm{AP}}_{\mathrm{prop}}$ (−9.4±1.72  mV, N=5) [Fig. 2(b)]. Furthermore, the decrease in ${\mathrm{AP}}_{\mathrm{TECC}}$ duration (−159±17  μs, N=7) was also statistically similar (p=0.9412) to that of ${\mathrm{AP}}_{\mathrm{prop}}$ (−157±27  μs, N=5) [Fig. 2(b)]. At an IR light power of 13.1 mW, despite more pronounced inhibition in the AP amplitude than at 7.1 mW, the inhibition on amplitude as well as duration between ${\mathrm{AP}}_{\mathrm{TECC}}$ and ${\mathrm{AP}}_{\mathrm{prop}}$ was statistically similar [Fig. 2(d)]. Thus, IR light-mediated inhibition of the AP waveforms is similar for both types of APs despite the fact that ${\mathrm{AP}}_{\mathrm{TECC}}$ typically took off ∼20  mV above the resting membrane potential whereas ${\mathrm{AP}}_{\mathrm{prop}}$ started near the resting level.

### 3.3 IR light Pulses Induced a Faster Rising in ${\mathrm{AP}}_{\mathrm{prop}}$

**Fig. 3:**
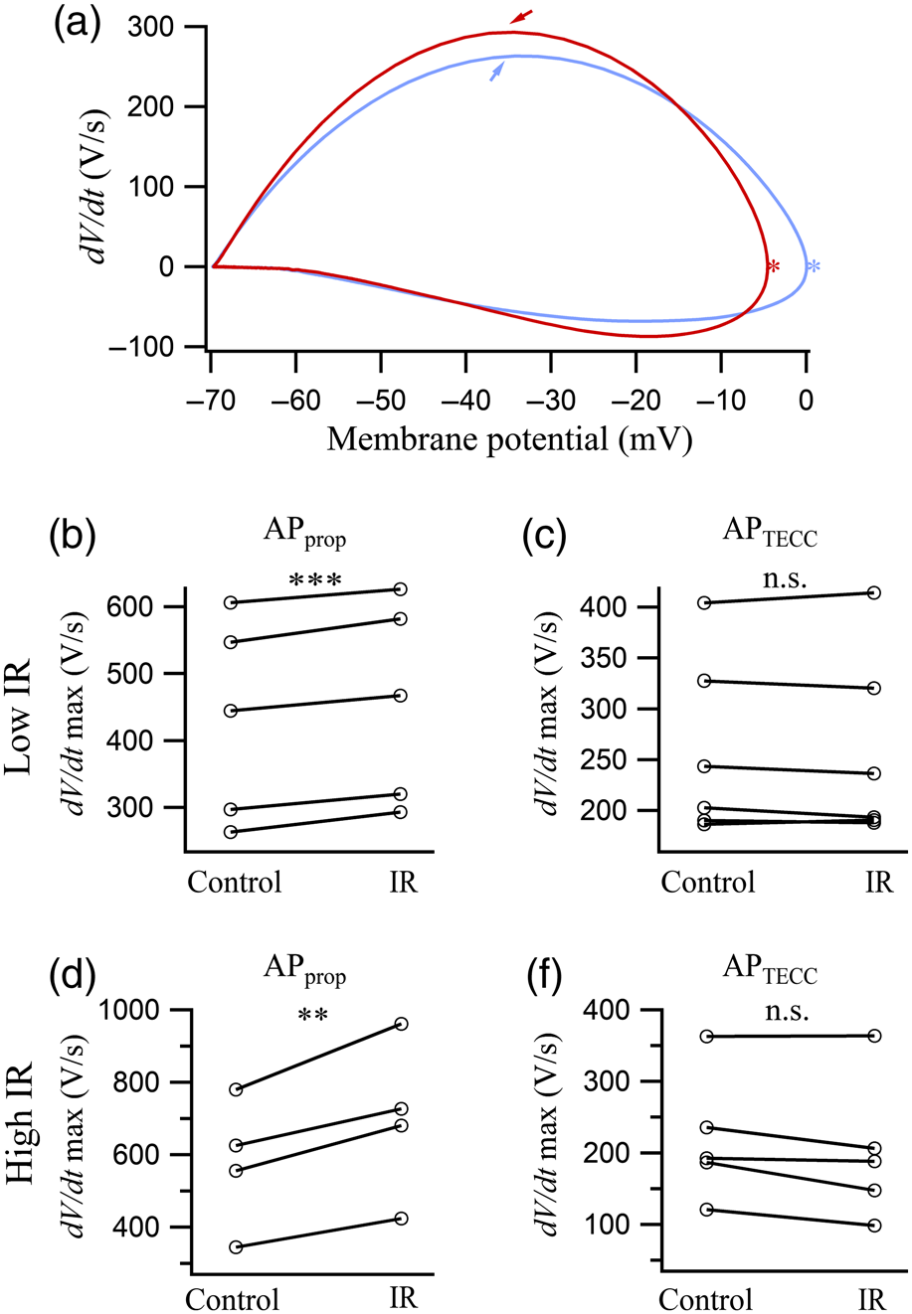
IR light pulses increased the maximum of dV/dt of the ${\mathrm{AP}}_{\mathrm{prop}}$. (a) Phase plot of a pair of ${\mathrm{AP}}_{\mathrm{prop}}$ with (red) and without (blue) IR light. Arrows indicate the maximum of dV/dt. Stars indicate the amplitude of the ${\mathrm{AP}}_{\mathrm{prop}}$. (b) The maximum of dV/dt of the ${\mathrm{AP}}_{\mathrm{prop}}$ under IR light illumination (N=5) exhibited a significant increase of 7±1.7% (p=0.0006) compared to the control values. (c) The maxima of dV/dt of the ${\mathrm{AP}}_{\mathrm{TECC}}$ under control and IR light illumination conditions (N=6) did not differ statistically (p=0.5620). (d) The maxima of dV/dt of the ${\mathrm{AP}}_{\mathrm{prop}}$ under 13.1-mW IR light illumination (N=4) exhibited an increase of 21%±1.6% (p=0.0011) compared to the control values. (e) The maxima of dV/dt of the ${\mathrm{AP}}_{\mathrm{TECC}}$ under control and IR light illumination at 13.1-mW conditions (N=5) did not differ statistically (p=0.0656).

In addition to the AP amplitude and duration, we observed a faster rise in the ${\mathrm{AP}}_{\mathrm{prop}}$ [Figs. 1(k) and 1(l)] but not in the ${\mathrm{AP}}_{\mathrm{TECC}}$ [Figs. 1(i) and 1(j)] during IR light illumination. To examine this difference quantitatively, the maximum of the first derivative (dV/dt) of the APs during the rising phase was compared. The change was best visualized in the phase plots of the control and IR light suppressed ${\mathrm{AP}}_{\mathrm{prop}}$ [Fig. 3(a)], with the latter exhibiting a higher maximum (arrows) but a lower peak amplitude (stars). The higher maximum for IR light (7.1 mW) suppressed ${\mathrm{AP}}_{\mathrm{prop}}$ was consistently observed in five preparations. The averaged percentage increase of dV/dt maximum was statistically significant [7%±1.7%, N=5, p=0.0006; Fig. 3(b), indicated by ***]. This increase in dV/dt maximum went up to 21±1.6% (N=4, p=0.0011) with 13.1 mW of IR light illumination [Fig. 3(d)]. For the ${\mathrm{AP}}_{\mathrm{TECC}}$, the averaged percentage change in dV/dt maximum was not statistically different from zero for both the 7.1-mW IR light pulses [N=5, p=0.5620; Fig. 3(c), indicated by n.s.] and the 13.1-mW IR light pulses [N=5, p=0.0656; Fig. 3(e)].

### 3.4 Synaptic Outputs Downstream to APs Blocked and Suppressed by IR Light

**Fig. 4:**
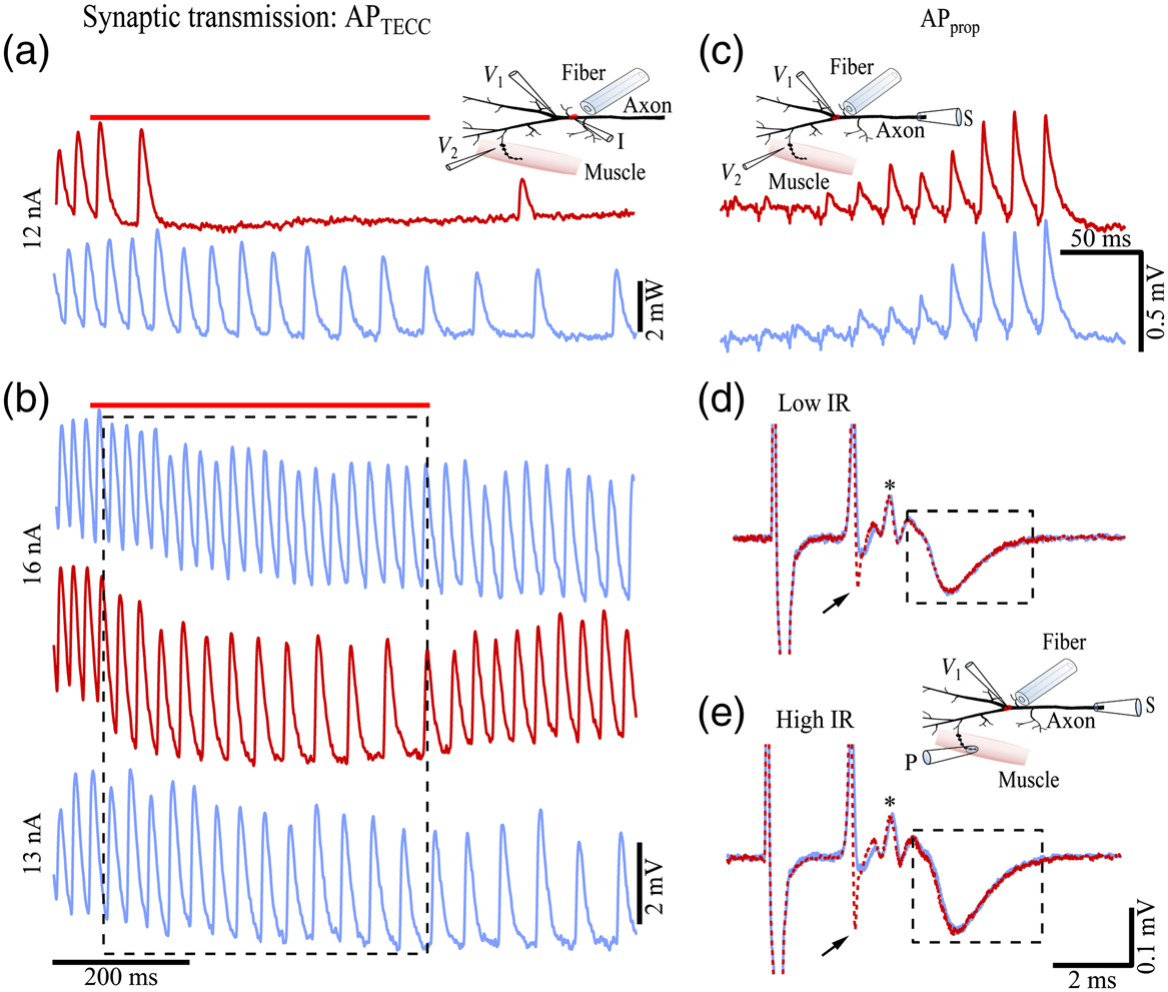
Effects of inhibiting ${\mathrm{AP}}_{\mathrm{TECC}}$ and ${\mathrm{AP}}_{\mathrm{prop}}$ on synaptic transmission. (a) IPSP trains recorded from a muscle cell ($V_{2}$ electrode in the inset) with (red) and without (blue) 7.1-mW power IR light illumination on the axon. The low-frequency ${\mathrm{AP}}_{\mathrm{TECC}}$ firing and the downstream IPSPs, evoked by a 12-nA current step, were blocked during the later period of IR light illumination. (b) IPSP trains at higher frequencies recorded from the same muscle cell as in (a), evoked by 16 (upper and middle traces) and 13 nA (bottom trace) steps. During the IR light irradiation, the amplitude of the individual IPSPs increased while the frequency decreased (middle red trace versus upper blue trace) due to the decreased ${\mathrm{AP}}_{\mathrm{TECC}}$ firing frequency caused by IR light illumination (7.1 mW). The IPSP amplitudes of the middle red trace within the dashed box are comparable to a control trace of comparable frequency (bottom blue trace). Red bars above (a) and (b) indicate the timing of the IR light pulses. (a) and (b) Share the same time calibration. (c) EPSPs ($V_{2}$ electrode in the inset) evoked by ${\mathrm{AP}}_{\mathrm{prop}}$ with (red) and without (blue) 7.1-mW power of IR light illumination on the axon. (d) and (e) Macro-patch recordings (P electrode in the inset) of the end-plate currents, evoked by ${\mathrm{AP}}_{\mathrm{prop}}$, with (red) and without (blue) 7.1 mW (d) and 13.1 mW (e) power of IR light illumination at the main branching point of the axon. Traces are averages of 60 trials. Arrows indicate the coupling potentials between $V_{1}$ and P electrodes. Stars indicate the APs at the presynaptic terminals.

Finally, we examined whether the block and suppression of APs by localized IR light illumination would result in changes in synaptic function. Synaptic function was monitored by an intracellular electrode placed in muscle cells >700  μm from the IR light illumination site, i.e., around the axon branching point [Figs. 4(a) and 4(c) insets]. This separation minimized the transfer and diffusion of the IR-induced localized heat of the axon to the muscle being recorded^19^ (Fig. S1). The traces in Fig. 4(a) showed the inhibitory postsynaptic potentials (IPSPs) evoked by low-frequency ${\mathrm{AP}}_{\mathrm{TECC}}$ firing with a 12-nA current step injected into the axon. IR light (7.1-mW power) blocked all IPSPs except for the first two, presumably by blocking the corresponding ${\mathrm{AP}}_{\mathrm{TECC}}$ in the axon. The upper and middle traces in Fig. 4(b) represented the IPSPs evoked by high-frequency ${\mathrm{AP}}_{\mathrm{TECC}}$ firing with a 16-nA current step. In this case, the IPSPs occurred at a higher frequency without IR light illumination [Fig. 4(b) upper blue trace] than those with IR light [Fig. 4(b) middle red trace]. While the IR light illumination reduced the IPSP frequency, individual IPSPs appeared larger [Fig. 4(b) middle red trace]. Since the IPSP amplitude is highly sensitive to the AP firing frequency due to short-term synaptic plasticity, we sought to control this variable by selecting control IPSPs evoked by a lower frequency ${\mathrm{AP}}_{\mathrm{TECC}}$ firing [Fig. 4(b) bottom blue trace; 13 nA] such that the IPSP frequency was comparable to that recorded with IR light illumination [Fig. 4(b) middle red trace]. The similarity between the IPSP amplitude and duration of the middle and bottom traces within the dashed box suggested that the differences in the IPSP waveforms between these upper and middle traces were attributable to the changed ${\mathrm{AP}}_{\mathrm{TECC}}$ firing frequency. In other words, the suppressed ${\mathrm{AP}}_{\mathrm{TECC}}$ by IR light pulses at the AP initiation site most likely recovered their normal waveform when they arrived at the terminals of this muscle cell. Similar observations were obtained in three additional preparations.

The postsynaptic potentials evoked by ${\mathrm{AP}}_{\mathrm{prop}}$ were also examined. Figure 4(c) shows the EPSPs resulting from a train of 10 ${\mathrm{AP}}_{\mathrm{prop}}$ induced with a suction electrode. The AP firing response and IR light illumination protocols were the same as those shown in Fig. 1(b) (see inset for recording configuration). The EPSPs exhibited no difference between the control (blue) and IR light illuminated (red, 7.1 mW) traces, which indicated that the suppressed ${\mathrm{AP}}_{\mathrm{prop}}$ recovered after emerging from the localized IR light exposure area. [The lower power level used in this report, 7.1 mW, was sufficient to suppress the synaptic output if the IR light illumination was aimed directly at the recorded muscle cell (Fig. S2 in the Supplementary Material).]

### 3.5 Simultaneous Monitoring of Presynaptic APs and Postsynaptic Responses at the Synapses of the IR Light Inhibited Axon

While the IPSP recordings in Figs. 4(a)–4(c) suggest that the suppressed APs recovered their waveform by the time they reached the presynaptic terminals, the actual AP waveform in the terminals was not monitored. To examine the AP waveform in the terminal, the macro-patch technique was used [Figs. 4(d) and 4(e) inset]. In this configuration, a macro-patch pipette was placed on a cluster of terminal varicosities, visualized after the axon was injected with Alexa 568. Figure 4(d) shows an example of such recording with (dotted red) and without (blue) IR light illumination on the axon. The first transient (arrow) following the large stimulation artifact represented the coupling potential between the ${\mathrm{AP}}_{\mathrm{prop}}$ recording electrode ($V_{1}$) and the macro-patch electrode (P). This transient exhibited a larger negative inflection with IR light illumination because it approximated the time derivative of the ${\mathrm{AP}}_{\mathrm{prop}}$ recorded in the $V_{1}$ electrode. This was consistent with the observation that the ${\mathrm{AP}}_{\mathrm{prop}}$ recorded during the IR light illumination exhibited accelerated repolarization [Figs. 1(k) and 1(l)]. The smaller, positive transient [Figs. 4(d) and 4(e) *] represented the APs in the presynaptic terminals.^41^ The inflection within the dashed box is the EPSC recorded by the macro-patch pipette. The terminal APs and the EPSCs overlap well between the control (blue) and IR light illuminated (dotted red) traces, indicating the recovery of the suppressed ${\mathrm{AP}}_{\mathrm{prop}}$. This close overlap of the presynaptic AP transients (star) and the EPSCs was also observed when the IR light power was increased to 13.1 mW [Fig. 4(e)], although the inhibition in the AP amplitude and duration was more pronounced at this power level [Fig. 1(I)]. The stronger suppression of the ${\mathrm{AP}}_{\mathrm{prop}}$ for an IR light power of 13.1 mW was indicated by the lower minimum of the coupling potentials [Fig. 4(e) arrow] since the repolarization was further accelerated by the stronger IR light power [Fig. 1(l)]. Similar observations were obtained in two additional preparations. Thus, both the intracellular recordings from the muscle cells and the macro-patch recordings of the terminals suggested that the AP waveforms were likely to have recovered after they emerged from the illuminated area where their amplitude and duration were suppressed significantly but not blocked.

## 4 Discussion

In this study, we investigated the effects of INI of ${\mathrm{AP}}_{\mathrm{TECC}}$ and ${\mathrm{AP}}_{\mathrm{prop}}$ in motor axons as well as their corresponding synaptic outputs. Recordings from the motor axons and muscle cells provided a precise correlation between the inhibition of the APs and the synaptic output from the same axons for the first time to the best of our knowledge. We found that IR light pulses at a lower power of 7.1 mW can completely and reversibly terminate the ${\mathrm{AP}}_{\mathrm{TECC}}$ firing at low frequencies. However, IR pulses of the same power level and even higher power level (13.1 mW) could only suppress but not completely block the ${\mathrm{AP}}_{\mathrm{prop}}$ nor the ${\mathrm{AP}}_{\mathrm{TECC}}$ under a strong depolarizing drive. The suppression in AP amplitude and duration was statistically comparable between the ${\mathrm{AP}}_{\mathrm{prop}}$ and the ${\mathrm{AP}}_{\mathrm{TECC}}$. In contrast, the maximum of the dV/dt of the ${\mathrm{AP}}_{\mathrm{prop}}$ increased significantly upon illumination, at both 7.1 and 13.1 mW, while the same parameter calculated from ${\mathrm{AP}}_{\mathrm{TECC}}$ remains unchanged. Blocking of the ${\mathrm{AP}}_{\mathrm{TECC}}$ initiation led to a corresponding block of the postsynaptic potentials, while suppression of the waveforms of either ${\mathrm{AP}}_{\mathrm{TECC}}$ or ${\mathrm{AP}}_{\mathrm{prop}}$ did not change the postsynaptic responses measured at a distance. These observations suggest that the suppressed APs of the motor axons can resume their waveforms after passing the localized IR light illumination site. Thus, to effectively modulate the motor outputs, the IR light illumination parameters need to be optimized for individual preparations or applications.

### 4.1 Mechanisms Underlying the IR-Mediated Inhibition

While the IR-mediated inhibition of the amplitude and duration of the ${\mathrm{AP}}_{\mathrm{TECC}}$ and the ${\mathrm{AP}}_{\mathrm{prop}}$ was quantitatively similar, IR light irradiation did significantly accelerate the rising phase of the ${\mathrm{AP}}_{\mathrm{prop}}$ (Fig. 3). Previous analyses have suggested that the first derivative of an AP during its rising phase approximates the time course of the sodium current ($I_{\mathrm{Na}}$) during that period.^44^ This increase in dV/dt was not as consistently observed in ${\mathrm{AP}}_{\mathrm{TECC}}$, which can be explained by the following two reasons. First, the ${\mathrm{AP}}_{\mathrm{TECC}}$ firing threshold during intracellular current steps fell typically between −35 and −45  mV. In contrast, the ${\mathrm{AP}}_{\mathrm{prop}}$ typically took off from a level around −70  mV and reached threshold in less than 100 ms. Second, the ${\mathrm{AP}}_{\mathrm{TECC}}$ used for this analysis was chosen from the end of the IR light illumination period, which corresponded to ∼600  ms after the onset of the current step. Both the depolarized level from which the ${\mathrm{AP}}_{\mathrm{TECC}}$ was initiated and the prolonged period of depolarization could have led to a significant accumulation of sodium channel inactivation. As a result, the effect of elevated temperatures on the accelerated sodium channel opening might not be detectable in the ${\mathrm{AP}}_{\mathrm{TECC}}$. In contrast to the AP rising phase, during which the sodium current was the dominant current, the time window in which the AP amplitude and duration were measured also involved potassium channel activation and sodium channel inactivation. Numerical simulations that take into account rate constants of these channels and their temperature dependence^22^^,^^23^ are further needed to offer a mechanistic explanation for the observation that IR-mediated inhibition on the amplitude and duration ${\mathrm{AP}}_{\mathrm{TECC}}$ and ${\mathrm{AP}}_{\mathrm{prop}}$ is statistically similar.

### 4.2 Comparison with Previous Studies and Significance

Motor outputs resulted from the IR-mediated inhibition of the peripheral nerves have not been studied as extensively as the IR-mediated motor excitation.^11^^–^^13^^,^^45^^–^^54^ In a previous study,^20^ it was shown that the IR light irradiation on a rat sciatic nerve could suppress the electrically evoked electromyography (EMG) signals of the gastrocnemius muscles. Since the sciatic nerve compound action potentials (CAPs) were not reported in the study, it is not directly possible to correlate the inhibition of the nerve CAPs and the muscle EMG signals. The same study^20^ also showed that the IR light on an Aplysia unmyelinated nerve reduced the CAPs amplitude and the muscle contraction force, but these parameters were not recorded in the same preparation. The CAP amplitude measured from a peripheral nerve represents the sum of APs from individual axons of the nerve. An IR-mediated reduction in the CAP amplitude could be due to the block of APs in some of the axons and the suppression of the APs in others. As suggested by data presented here, it is likely that the recovery of the suppressed APs could happen in healthy preparations as they propagate toward the presynaptic terminals. This possibility has been suggested in a simulation study.^22^ Thus, INI of the motor output cannot be precisely predicted from the magnitude of the inhibition of the CAP amplitude. Precise and reliable inhibition of muscle activities by localized IR light irradiation on axons, especially for long axons or myelinated axons with large safety factors, needs to consider a combination of factors, such as the irradiation location, area, and the power level. For example, aiming the IR light near the muscle may be more efficient over other locations, for several reasons.^55^ First, if the location of the inhibited ${\mathrm{AP}}_{\mathrm{prop}}$ is near the axonal terminals, the suppressed APs may not have enough propagation distance to regain their normal shape. Second, IR light irradiation near the target muscle may have the added benefit of inhibiting synaptic function and muscle cells directly.^36^^,^^56^ This approach may have the advantage of expanding the direct reach of IR light on the Ca^2+^ channels in the presynaptic terminals, the molecular processes regulating synaptic transmission, and the generation of APs in muscle cells. Other technical innovative approaches such as combining IR light with other modalities^34^ may also improve the efficiency and precision of IR-mediated motor modulation.

Our observation that the IR light pulses terminated the ${\mathrm{AP}}_{\mathrm{TECC}}$ firing at low frequencies but not those at high firing frequencies nor the ${\mathrm{AP}}_{\mathrm{prop}}$ suggests that the physiological and anatomical “contexts” of an AP are important to evaluate or predict the impact of the IR-mediated modulation. When IR light pulses are applied to complex neuronal networks such as mammalian cortexes,^3^^,^^6^^–^^9^ the IR light inhibition of ${\mathrm{AP}}_{\mathrm{TECC}}$ could serve as a basic model for understanding the impact of IR light on neuronal soma and on the axon initial segment. Assuming neurons in a network are under various levels of synaptic drive, our results suggest that neurons under weak excitatory drive will be inhibited disproportionally compared to those under strong excitatory drive. Since the function of a neuronal network is mainly defined by the frequency and the number of APs emerging from the network, our results offer a rule on how IR light may bias the output of a network. Specifically, IR light irradiation of a complex network will preferentially inhibit components with weak excitatory drive while favoring elements in the network with strong excitatory drive. Morphological factors are also important to consider when a complex network is exposed to IR light irradiation. It has been shown that the size of axons can influence their sensitivity to IR-mediated inhibition, with thinner axons being more sensitive.^21^ Finally, the direct effects of IR light on synaptic function remain to be incorporated. Thus, IR irradiation on a complex network—composed of neurons with diverse morphology and physiological states—does not influence all components equally. Our results advance our understanding of basic “bias” of IR light-mediated modulation, which poses an important step toward being able to predict IR-mediated network outcomes.

## 5 Conclusion

We used the crayfish neuromuscular preparation as a model to study the 2-μm IR light-mediated inhibition of the axonal excitability under different physiological contexts and the corresponding synaptic outputs. Results here provided for the first time a detailed demonstration of the inhibitory modulation of the peripheral neuromuscular system mediated by localized IR light illumination. We showed that IR light irradiation suppressed the AP amplitude and duration similarly for ${\mathrm{AP}}_{\mathrm{TECC}}$ and ${\mathrm{AP}}_{\mathrm{prop}}$. IR light irradiation generated a significant increase in the maximum of dV/dt in ${\mathrm{AP}}_{\mathrm{prop}}$ but not in ${\mathrm{AP}}_{\mathrm{TECC}}$, which could be attributed to an accelerated ${\mathrm{Na}}^{+}$ channel activation upon IR light illumination. While a certain IR light power was sufficient to block ${\mathrm{AP}}_{\mathrm{TECC}}$ firing evoked by a weak excitatory drive, it failed to completely block ${\mathrm{AP}}_{\mathrm{prop}}$ and ${\mathrm{AP}}_{\mathrm{TECC}}$ evoked by a strong current drive. Recordings from muscle cells showed that the synaptic transmission was blocked only when the corresponding axonal APs were completely blocked. Changes in the frequency response of the synaptic transmission were observed when the axonal AP firing frequency was reduced by IR light. A suppression of the AP amplitude and duration did not lead to a reduction in synaptic output recorded in the muscle cell distant to the IR light illumination site. In fact, macro-patch recordings suggested that the ${\mathrm{AP}}_{\mathrm{prop}}$ recovered their waveforms in presynaptic terminals despite having been suppressed in the proximal part of the axon. The presented impacts of IR light inhibition on ${\mathrm{AP}}_{\mathrm{TECC}}$ and ${\mathrm{AP}}_{\mathrm{prop}}$ can be used as a reference and guidance for applications of IR light modulation technology in peripheral and central nervous systems.
