# Supplementary material for: Infrared inhibition impacts on locally initiated and propagating action potentials and the downstream synaptic transmission
Source: Neurophotonics. 2020 Oct 14;7(4):045003. doi: 10.1117/1.NPh.7.4.045003 (PMC7554448; doi:10.1117/1.NPh.7.4.045003)
Supplement: Supplementary file 1 [file NPh_007_045003_SD001.docx]

**Infrared inhibition impacts on locally initiated and propagating action potentials and the downstream synaptic transmission**

Xuedong Zhu^a,b,c^, Jen-Wei Lin^d^, Michelle Y. Sander^a,b,c,e,f,*^

^a^Department of Biomedical Engineering, Boston University, 44 Cummington Mall, Boston, MA 02215, USA

^b^Neurophotonics Center, Boston University, 24 Cummington Mall, Boston, MA, 02215, USA

^c^Photonics Center, Boston University, 8 St. Mary’s Street, Boston, MA 02215, USA

^d^Department of Biology, Boston University, 5 Cummington Mall, Boston, MA 02215, USA

^e^Department of Electrical and Computer Engineering, Boston University, 8 St. Mary’s Street, Boston, MA 02215, USA

^f^Division of Materials Science and Engineering, Boston University, 15 St. Mary’s Street, Brookline, MA 02446, USA

*Address for correspondence:

Michelle Sander

Associate Professor, Boston University

Electrical and Computer Engineering, BU Photonics Center,

Division of Materials Science and Engineering

Department of Biomedical Engineering

8 St. Mary’s Street PHO 534

Phone: 617-358-0505

Email: msander@bu.edu

**SUPPLEMENTARY MATERIAL**

**S1 IR-induced temperature rise distribution** In this study, the saline was circulated at a speed of 1 – 1.5 ml/min and the IR light was delivered via a 50 µm optical fiber covering an area about 50 µm × 100 µm. So even though the exposure time is longer than the thermal relaxation time of the saline, the temperature drops significantly outside of the illuminated area, consistent with other reports (see refs. 30 and 32 in the manuscript). We have made some preliminary assessments with open patch pipettes (see ref. 19 in the manuscript) and show one example in the following Fig. S1. The solid line is the temperature transient recorded from the center of the illumination area and the dashed line is recorded about 50 – 100 µm from the edge of the illuminated area. The temperature increase away from the directly illuminated area (off center) is about 20% of the peak temperature recorded. Thus, we can regard the light as localized in that thermal diffusion does not lead to any significant temperature contributions outside of the fiber spot size. In this report, postsynaptic recordings from muscle cells and macro-patch recordings of the synaptic terminals were obtained > 700 µm distal to the IR light illuminated site is therefore well outside of region with significant temperature rise.

**S2 Direct illumination of the muscle** Though we showed that the synaptic transmission measured at a distance was unaltered, IR light illumination directed at recorded muscle fibers at 7.1 mW did suppress postsynaptic potentials (Fig. S2). The excitatory postsynaptic potentials (EPSPs) were evoked by 11 consecutive propagating APs with the similar protocol as depicted in Fig. 1(b) in the manuscript. With 7.1 mW power IR light illumination on the recorded muscle cell, the EPSPs (red trace) were significantly smaller than the control (blue trace). The direct inhibition of muscle activities by IR light pulses has been reported (see refs. 36 and 56 in the manuscript).

**Supplementary figures**


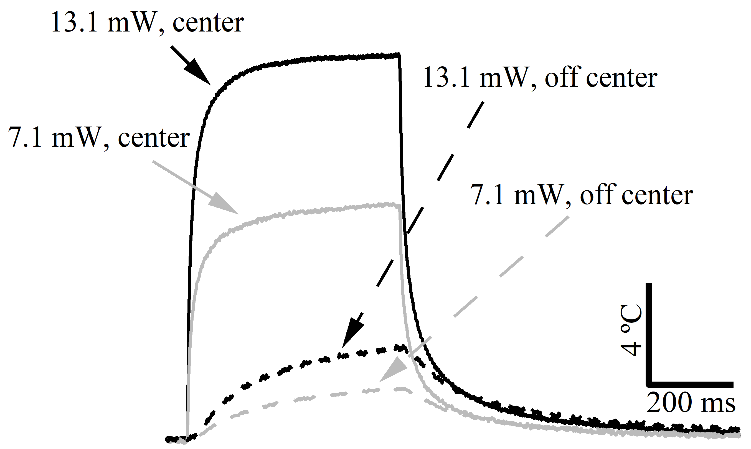


**Fig. S1** Spatial decay of IR light induced temperature transients. The temperature rise was monitored with open patch technique, which was first positioned at the center of the illuminated area and then horizontally displaced by 100 µm from the edge of illuminated area, 150 to 200 µm from the center. This measurement and all our experiments were performed with continuous circulation of saline at a speed of 1 – 1.5 ml/min. In this report, postsynaptic recordings with intracellular electrodes and macro-patch recordings of the terminals were obtained > 700 µm distal to the IR light illumination site, which was well outside the region with significant temperature rises. The duration of the IR light pulses was 500 ms. Traces were averaged over 3 – 5 trials.


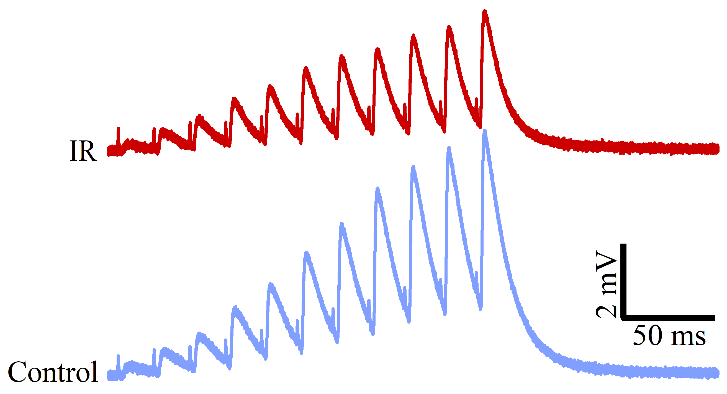


**Fig. S2** EPSPs were suppressed (red trace) by direct illumination on the muscle with 500 ms IR light pulses at 7.1 mW power. Traces were averaged over 40 trails.


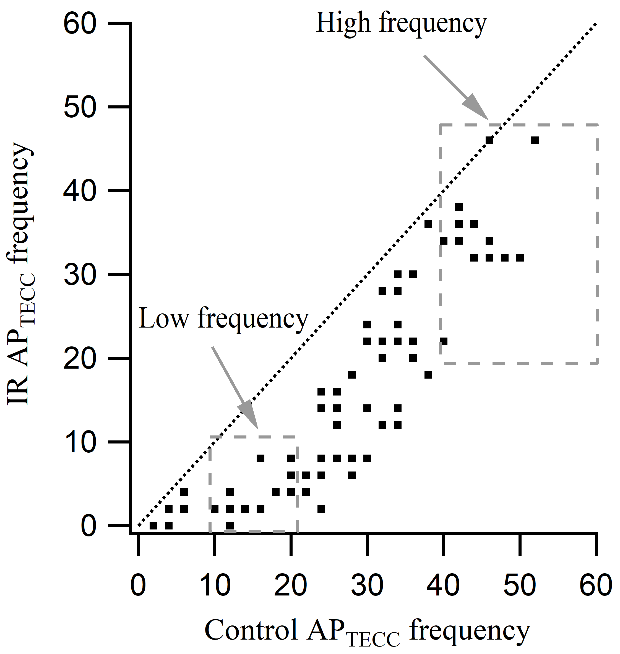


**Fig. S3** Illustration of the distribution of firing frequencies of AP_TECC_ with 500 ms IR light pulses at 7.1 mW power in a typical experiment. The AP firing frequencies measured during the IR light illumination were plotted against those measured during the same time window but without IR light illumination. Each data point represents responses evoked by the same current step. All data points were collected from the same axon and under the same IR light power. Data point scattering reflects trial-to-trial variations as well as systematic increases in injected current. The straight line represents the identity line and deviations from it highlight more pronounced inhibition in the low frequency region than in the high frequency region. The low frequency range is defined from 10 – 20 Hz during the control period while the high frequency range is from 40 – 60 Hz.
